# Recent advances in biological pumps as a building block for bioartificial hearts

**DOI:** 10.3389/fbioe.2023.1061622

**Published:** 2023-01-20

**Authors:** Sunita Brimmer, Pengfei Ji, Aditya K. Birla, Sundeep G. Keswani, Christopher A. Caldarone, Ravi K. Birla

**Affiliations:** ^1^ Laboratory for Regenerative Tissue Repair, Texas Children’s Hospital, Houston, TX, United States; ^2^ Center for Congenital Cardiac Research, Texas Children’s Hospital, Houston, TX, United States; ^3^ Division of Congenital Heart Surgery, Texas Children’s Hospital, Houston, TX, United States; ^4^ Department of Surgery, Baylor College of Medicine, Houston, TX, United States; ^5^ Division of Pediatric Surgery, Department of Surgery, Texas Children’s Hospital, Houston, TX, United States

**Keywords:** tissue engineering, heart, pump, cardiomycoyte, bioreactor

## Abstract

The field of biological pumps is a subset of cardiac tissue engineering and focused on the development of tubular grafts that are designed generate intraluminal pressure. In the simplest embodiment, biological pumps are tubular grafts with contractile cardiomyocytes on the external surface. The rationale for biological pumps is a transition from planar 3D cardiac patches to functional biological pumps, on the way to complete bioartificial hearts. Biological pumps also have applications as a standalone device, for example, to support the Fontan circulation in pediatric patients. In recent years, there has been a lot of progress in the field of biological pumps, with innovative fabrication technologies. Examples include the use of cell sheet engineering, self-organized heart muscle, bioprinting and *in vivo* bio chambers for vascularization. Several materials have been tested for biological pumps and included resected aortic segments from rodents, type I collagen, and fibrin hydrogel, to name a few. Multiple bioreactors have been tested to condition biological pumps and replicate the complex *in vivo* environment during controlled *in vitro* culture. The purpose of this article is to provide an overview of the field of the biological pumps, outlining progress in the field over the past several years. In particular, different fabrication methods, biomaterial platforms for tubular grafts and examples of bioreactors will be presented. In addition, we present an overview of some of the challenges that need to be overcome for the field of biological pumps to move forward.

## Introduction

There is a chronic shortage of donor hearts in both the adult and pediatric population, with significant challenges in both patient populations (Evers et al., 2011; Alyaydin et al., 2020; Jiritano et al., 2020; Lin and Fang, 2020; Malik et al., 2020). In both the adult and pediatric population, the quality of life is comprised in heart transplant recipients, due to the need to be on lifelong immunosuppression in both the adult and pediatric patient populations (Asleh et al., 2018; Gale et al., 2019; Heeney et al., 2019; Hussain et al., 2021; Mylonas et al., 2022; Nassetta et al., 2022). In the pediatric population, in addition to the challenges with lifelong immunosuppression, there are very unique challenges associated with retransplantation; there is a high risk for retransplant of the heart after a decade of initial transplant due to growth of the pediatric patient (Greenleaf et al., 2016). While the burden of a single transplant on a pediatric patient is challenging, confounding this problem with the need for a retransplant to match the metabolic needs of the growing patients, which makes this situation even more challenging.

The holy grail in the field of cardiac tissue engineering is the ability to bioengineer a complete bioartificial heart (Birla and Williams, 2020). The ability to bioengineer transplantable hearts that are not rejected by the patient will be a life option for thousands of patients across the globe annually (Virani et al., 2021). The human heart is a very complex organ (Docherty, 2005) and there are numerous challenges in accomplishing this milestone. To tackle this challenge, there are many different and parallel strategies being tested. The field of cardiac tissue engineering has been broadly divided into two categories. The first approach, and the more ambitious, is focused on strategies to bioengineer a fully functional transplantable heart (Ott et al., 2008; Lu et al., 2013; Yasui et al., 2014; Hogan et al., 2015; Tao et al., 2015; Noor et al., 2019). This approach is the most ideal from a clinical standpoint and once successful, will lead to an off the shelf option for heart transplant patients. Clearly, this approach is challenging, and many scientific and technological milestones must be met before accomplishing this goal. The approaches for whole heart bioengineering have also been very diverse, ranging from the use of acellular matrices (Ott et al., 2008; Lu et al., 2013) to the use of recent advance in bioprinting methods (Noor et al., 2019).

The second approach is focused on bioengineer components of the hearts, divided based on function to include contractile tissue which include heart muscle (Hogan et al., 2015; Tao et al., 2017; Morrissette-McAlmon et al., 2018; Pena et al., 2018; Rodrigues et al., 2018; Rosellini et al., 2018; Tao et al., 2018; Tijore et al., 2018; Turnbull et al., 2018; Valarmathi et al., 2018; Vozzi et al., 2018; Wu and Guo, 2018; Abbasgholizadeh et al., 2020; Birla, 2020a), ventricles (Yildirim et al., 2007; Lee et al., 2008; Patel and Birla, 2016; Patel et al., 2016; Patel et al., 2017; Li et al., 2018; MacQueen et al., 2018; Patel and Birla, 2018; Lee et al., 2019; Birla, 2020b) and the biological pumps (Mohamed et al., 2015), the electrical tissue to include the Purkinje networks (Tracy et al., 2020) and the vasculature (Marsano et al., 2013; Mehrabi et al., 2020). There are numerous examples in the recent literature that show different technologies that have been adapted for the use in bioengineering cardiac tissue with excellent review articles covering recent state of the art (Williams and Birla, 2020). There are numerous reasons for bioengineering cardiovascular tissue. First, individual components can be assembled to form functional transplantable hearts, a parallel streety to bioengineering whole hearts. Second, in cases of acute myocardial dysfunction, where a heart transplant is not needed, bioengineered heart muscle tissue could be used to support myocardial dysfunction. Such a therapeutic strategy can be used in combination with currently used beta blockers and angiotensin converting enzyme (Piacentini et al., 2007) inhibitors. Third, bioengineer cardiovascular tissue can be developed and used for high throughput drug screening and/or cardiotoxicity testing. Forth, the lesions learnt in fabricating functional cardiovascular tissue can be translated to whole heart bioengineering.

While there are two major categories in the field of cardiac tissue engineering, as described earlier, there are other avenues that are being pursued. For example, there has been much interest in developing spheroids and other organoid models that can be used for high throughput drug screening (Hribar et al., 2015; Behroodi et al., 2020; Feng et al., 2020; Hong and Song, 2021). These spheroids and organoids can be viewed as a small conglomerate of cells, that have been fabricated in a 3D configuration. The diameter of the spheroids is in the range of a couple hundred microns and contains a couple hundred cells per spheroid. These spheroids are being designed as potential tools for high throughput screening for cardiotoxicity testing as they can maintained in a 96-well or 384-well configuration (Hribar et al., 2015; Behroodi et al., 2020; Feng et al., 2020; Hong and Song, 2021).

The field of cardiac tissue engineering is vast and expanding and only a brief introduction has been presented in this article; an overview of the field of cardiac tissue engineering has been the focus of several recent review articles (Boroumand et al., 2021; Gisone et al., 2022; Roacho-Perez et al., 2022; Zhuang et al., 2022). In this article, we review recent advances in one specific aspect of the field, biological pumps. In recent years, there have been several excellent review articles in the field of biological pumps, though most of them have been very narrow in focus and covering cell sheet engineering methods (Sekine et al., 2012; Haraguchi et al., 2014; Sekine et al., 2016; Kobayashi et al., 2019). While cell sheet engineering has been a very important development and one that has moved the field of forward, recent work in this space has expanded to cover many different fabrication technologies. The purpose of this review article is to provide an overview of the field of biological pumps, to include cell sheet engineering and other recent methods in the field.

## What exactly are biological pumps?

The concept of a biological pump is not one that is obvious. This is in comparison to other areas in the field of cardiac tissue engineering, for example, like cardiac patches, that are easier to understand, have an extensive publication record (Cho et al., 2022) and a clear clinical application; for example, bioengineered cardiac patches are designed to increase the lost functional of heart muscle tissue after acute myocardial infarction (Jackman et al., 2015; Jackman et al., 2018). However, there are not very many publications related to biological pumps and the clinical applications are not obvious. Prior to a discussion of biological pumps, a brief overview of cardiac patches will provide the necessary framework. Cardiac patches are planer 3D tissue constructs fabricated by culturing contractile cardiomyocytes within a biologically active hydrogel or another biomaterial, Figure 2A. Functional integration at the host-material results coupling of the cardiomyocytes with the functional sites on the biomaterial fibers and support formation of functional 3D heart muscle. These tissue patches are contractile, and the functional performance is measured by quantification of the twitch force of contraction. The objective is to fabricate 3D cardiac patches that are similar in form and function to mammalian heart muscle tissue. Most of the field, if not all, is devoted to developing new technologies to bridge the gap between bioengineered and mammalian heart muscle tissue (Cho et al., 2022).

The transition from a 3D planar patch to a bioartificial heart is one that is faced with challenges, and not a simple jump from the former to the later. The transition is also not a linear, with several developmental milestones along the pathway. Several enabling technologies are critical to move this field forward, including advancements in valve bioengineering, development of vasculature and electrical conduction system and the fabrication of the complex geometry of left and right atrium and ventricles. The transition from 3D cardiac patches to whole hearts also represents a transition in functional metrics, from twitch force of contraction to left ventricle pressure, or in simpler terms, from contraction to intraluminal pressure.

The concept of biological pumps was developed to facilitate the transition from 3D cardiac patches to bioartificial hearts and provide an intermediate step in the developmental platform. The early thinking was to develop technology “along the way” or “during transition” that can provide insight into the building blocks required for whole heart bioengineering. The initial concept was to develop a 3D tissue construct that can transition from contractility as the key performance metric to intraluminal pressure, more indicative of the functional metrics necessary for whole hearts. During the initial stages of development, biological pumps were developed as a tool to understand the alignment and orientation of contractile cardiomyocytes with 3D scaffolds in a tubular configuration and the subsequent requirements to support “pressure generation” as opposed to contractility measures in 3D patches.

## Applications for biological pumps

The field of biological pumps initially evolved to support the transition from planar 3D patches to pulsatile pumps and as a transition to whole hearts. However, as the field of biological pumps developed, it became clear there was many applications for biological pumps as a standalone technology. Biological pumps are now being developed as Fontan pumps to provide active support during the Fontan circulation, as biological assist devices and for high throughput cardiotoxicity testing, all of which are discussed here.

The Fontan circulation is particularly relevant during the surgical management of congenital patients with hypoplastic left heart syndrome (HLHS) (Hoashi et al., 2020; Chowdhury et al., 2022). HLHS is a condition in which neonates are born with a missing or underdeveloped left ventricle (Roeleveld et al., 2018; Bejjani et al., 2021; Birla et al., 2022; Jacobs, 2022). This condition is fatal is not aggressively managed at the time of birth. Patients with HLHS are managed through a series of three very complex timed surgeries. The Norwood operation is typically performed 1–2 weeks after birth, the Glenn operation is typically performed 4–6 months after birth and the Fontan operation is performed 3–4 years after birth. During the Fontan operation, the inferior vena cava is separate from the heart and connected to the pulmonary artery using an inert Gore-Tex conduit (Roeleveld et al., 2018; Arunamata et al., 2020; Danton, 2021; King et al., 2022). This conduit only serves to provide a length of inert tubing to support blood flow and does not have a contractile function (Hoashi et al., 2020; Daley and d'Udekem, 2021; Lee et al., 2007; Ochiai et al., 2009; Perez-Caballero et al., 2022). However, replacing this inert conduct with a pulsatile biological pump will add additional functionality; the pulsatile activity of the pump can be used to support the flow of blood through the interior vena cava to the pulmonary artery.

A second potential application of biological pumps is a biological left ventricular assist device (LVADs). LVADs are mechanical pumps that are used to support failing hearts by pumping blood directly from the left ventricle to the aorta. These devices are used extensively as a bridge to transplantation and also as destination therapy, for long-term applications of LVADs to support failing hearts. However, LVADs have been faced with challenges. The interface between the LVAD and host heart is non-functional, as LVADs are mechanical devices with no biological components and do not functionally integrate with the host myocardium. In addition, while LVADs are used the patients has to be on anticoagulation therapy, with a unique set of challenges. A biological pump, once it meets the functional requirements, will be able to alleviate the challenges. Biological pumps can be used as biological LVAD and provide the functional support needed, and also provide a biological interface with the host tissue.

Biological pumps can also be used a functional *in vitro* assay for cardiotoxicity testing. These pumps can be fabricated and maintained in a 6-well configuration and be exposed to different pharmacological agents and the subsequent effect on intraluminal pressure assessed. This will provide a significant advantage over current animal models used to conduct these test as biological pumps can be maintained in culture for extended periods of time compared to hours when rodent hearts are used. In addition, as these biological pumps are fabricated using human iPSC-CMs, all ethical challenges related to animal testing is removed.

These applications are very important, and each can have a very significant implications that can impact the quality of care for heart failure patients. While the field of biological pumps has evolved significantly over the past decade, there are very few review articles highlighting this work, the focus of this article. This article will provide an overview of the current state of the art in biological pumps and provide insight into the scientific challenges that need to be overcome to move this field forward.

## Design criteria for biological pumps

The goal of biological pumps is to support left ventricle output from the human heart and must satisfy rigorous functional performance metrics. At minimum, any biological pump should generate intraluminal pressures in excess of 120 mmHg. In addition, the inner surface of the biological pump must be lined with endothelial cells while the outer layers of cardiomyocytes (CMs) must be thick and vascularized. The outer layer of musculature must be fabricated using billions of mature patient specific induced pluripotent stem cell derived cardiomyocytes (iPSC-CMs). The biological pump must withstand millions of repetitive contractions and therefore, has to be fabricated using mechanically strong and yet compliant materials. Unidirectional valves must be incorporated at both ends to support unidirectional blood flow. Synchronous contractions of the biological pumps with the host heart are also another challenge, one that will require both sensing the host contractions and functioning in a synchronized manner. While all these requirements are necessary for a biological pump that can be used in the human body, no such pump has been described in the published literature. However, in the next few sections, we provide an overview of recent advances in biological pumps, which can be viewed as intermediate development steps, on the way to the ideal biological pump.

## Methodology to bioengineer cell based biological pumps

**FIGURE 1:**
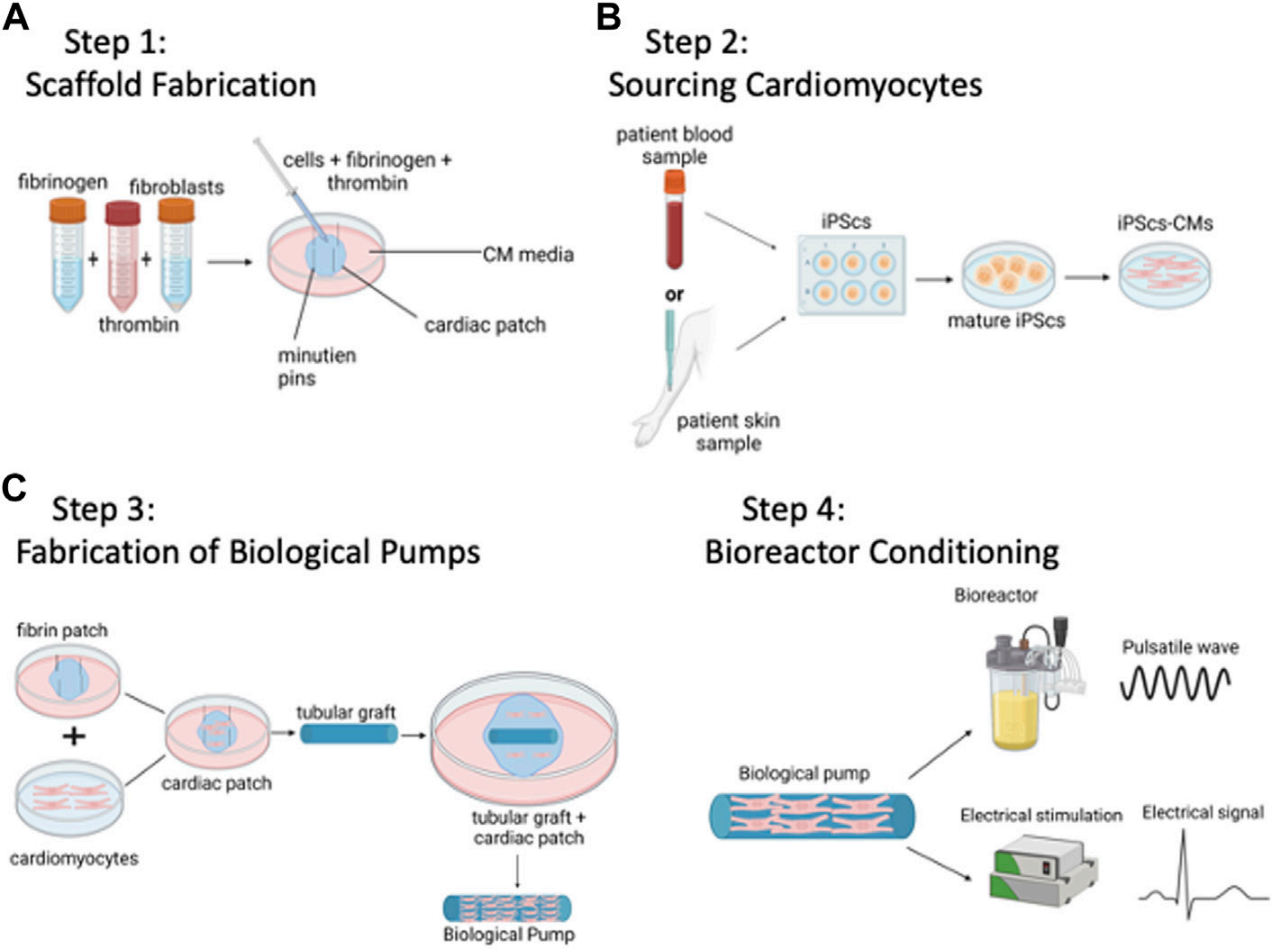
Method to Bioengineer Biological Pumps—**(A)** Step 1: Scaffold Fabrication. **(B)** Step 2: Sourcing Cardiomyocytes. **(C)** Step 3: Fabrication of Biological Pumps. **(D)** Step 4: Bioreactor Conditioning. Created with BioRender.com.

In this section, we describe a generic process to bioengineer biological pumps, Figure 1, while in a subsequent section, we describe published methods based on this process. Table 1 provides an overview of some of the recent methods to fabricate biological pumps.

**TABLE 1:** Models of biological pumps.

Year	Cells	Matrix	Fabrication Method	Pressure mmHg	Novelty	Ref
2006	NVRMs	Resected Rat Thoracic Aorta	Cell Sheet Engineering	5.9	Use of in vivo bioreactor for pump formation	Sekine et al. (2006)
2007	NVRMs	Fibrin Gel	Cell Sheet Engineering	0.15	Development of a method to create pumps using multiple cell sheets	Kubo et al. (2007)
2008	NVRMs	Chitosan	Self-organization	0.1	Use of self-organized heart muscle tissue as the functional platform	Birla et al. (2008b)
2009	NVRMs	Fibrin	*In vivo*	2.0	Use of *in vivo* biochamber to promote vascularization	Birla et al. (2009)
2010	NVRMs	Acellular Rat Aorta	Self-organization	0.05	Use of acellular rat aorta to support pump function	Evers et al. (2011)
2015	NVRMs	Acellular Goat Carotid Artery	Compaction of fibrin gel	Not Reported	Use of compaction of fibrin gel to support functional biological pumps	Mohamed et al. (2015)
2019	iPSC-CMs	Fibrin and Collagen	Cell Sheet Engineering	0.30	Use of iPSC-CMs coupled with cell sheet engineering	Tsuruyama et al. (2019)
2020	iPSC-CMs	Acellular Human Umbilical. Artery	Engineered Patches Wrapped Around Acellular Artery	0.90	Use of iPSC-CMs, engineered heart muscle tissue and acellular human umbilical artery in a single system.	Park et al. (2020)
2022	iPSC-CMs	Collagen	Printing	Not Reported	Novel use of extrusion-based printing to generate biological pumps	Bliley et al. (2022)
2022	iPSC-CMs	Fibrin Gel	Casting	0.30	Use of iPSC-CMs casted in a fibrin gel to generate pulsating pumps.	Kohne et al. (2022)

### Step 1: Scaffold fabrication

The goal is to fabricate a tubular scaffold that will serve as the template for the biological pump, Figure 1A. As with any material-based application, there are both biological and mechanical design constraints. Biologically, any tubular graft must be able to support the attachment, proliferation, and function of cardiomyocytes. Mechanically, the tubular graft must be able to withstand repetitive pulsatile contractions from the functional cardiomyocytes, in other words, functional as a pump without mechanical failure. Examples of materials have used are acellular vascular grafts from large animals, e.g., goat carotid arteries (Mohamed et al., 2015), or tubular grafts that have been bioengineered from polymeric scaffolds, with chitosan being one example (Evers et al., 2011). Generating a tubular scaffold is only one of several configurations that have been tested; another common configuration has been that of a left ventricle (Michas et al., 2022). In this case, the tubular scaffold is replaced with a cone shaped left ventricle, and again, populated with contractile cardiomyocytes. In this case, the goal is to bioengineer a functional human left ventricle, rather than a biological pump. In both cases, the applications are similar, and both biological pumps and left ventricles are designed to recapitulate the pumping function of the heart.

### Step 2: Sourcing of cardiomyocytes

The functional machinery or the powerhouse of the biological pumps, cardiomyocytes provide the repetitive and rhythmic contractions that drive the biological pumps, Figure 1B. During the early development of the field, CMs were isolated from neonatal ventricular rat cardiomyocytes (NVRMs) and used for initial proof of concept studies (Evers et al., 2011). These cells still prove to be an excellent choice for model development and optimization studies. The main advantages of using NVRMs are the ease of availability, low cost and the standard protocols for isolation and culture. This provides a significant advantage in the development of biological pumps as a large supply of cardiomyocytes is required to optimize the contractile platform. Recent studies have been focused on CMs derived from induced pluripotent stem cells, iPSC-CMs (Yildirim et al., 2007; Lee et al., 2008; Patel and Birla, 2016; Patel et al., 2016; Patel et al., 2017; Li et al., 2018; MacQueen et al., 2018; Patel and Birla, 2018; Lee et al., 2019; Tsuruyama et al., 2019). These methods lead to the development of patient specific therapies and/or technologies, often referred to as personalized medicine, and offer significant advantages in terms of patient specificity (Zhang et al., 2009; Hattori and Fukuda, 2012; Lian et al., 2013). However, challenges in working with iPSC-CMs include high costs, and technical expertise required. However, both iPSC-CMs and NVRMs continue to be instrumental in the field of biological pumps. One strategy that has been successfully adopted in this field is the use of NVRMs during early stages of model development, followed by iPSC-CMs once the models are developed to provide patient specific solutions.

### Step 3: Scaffold cellularization

The next step is coupling contractile CMs with the tubular scaffold to form functional biological pumps, Figure 1C. This is a challenging task as the number of CMs, seeding density, proportion of different cell types (CMs + fibroblasts) are important parameters that need to be optimized. In addition, cell viability, functional integration at the cell-material interface, intercellular connectivity and formation of electromechanical junctions are important functional parameters that also need measured. Examples of cellularization strategies used to populate tubular grafts with cells include direction injection of isolated CMs (Blan and Birla, 2008), placement of bioengineered heart muscle on the outer (Evers et al., 2011). In addition, a novel 2-stage cellularization strategy has been described (Patel et al., 2016), which consists of direct cell injection followed by anchoring of bioengineered heart muscle on the outer of the graft (Patel et al., 2016).

### Step 4: Bioreactor conditioning

Bioreactors are custom devices to simulate *in vivo* physiology during *in vitro* culture (Birla et al., 2007). The human heart is a complex organ with a myriad of signals that regulate function and physiology; these signals are essentials for cardiomyocytes to maintain differentiated phenotype and for regulation of cardiac output. Biological pumps need to be cultured in custom bioreactors to deliver coupled electromechanical stimulation of iPSC-CMs or NVRMs (Kashiwagi et al., 1998; Akhyari et al., 2002; Campbell and Chandra, 2006; Birla et al., 2007; Birla et al., 2008a; Clause et al., 2009; Salazar et al., 2015a; Salazar et al., 2015b; Abilez et al., 2018; Kaur et al., 2020). In addition, continuous media flow is important to meet the metabolic demands of the biological pumps (Kashiwagi et al., 1998; Akhyari et al., 2002; Campbell and Chandra, 2006; Khait et al., 2008a; Hecker et al., 2008; Clause et al., 2009; Hecker et al., 2009; Abilez et al., 2018; Kaur et al., 2020).

## Cell sourcing for biological pumps

**FIGURE 2:**
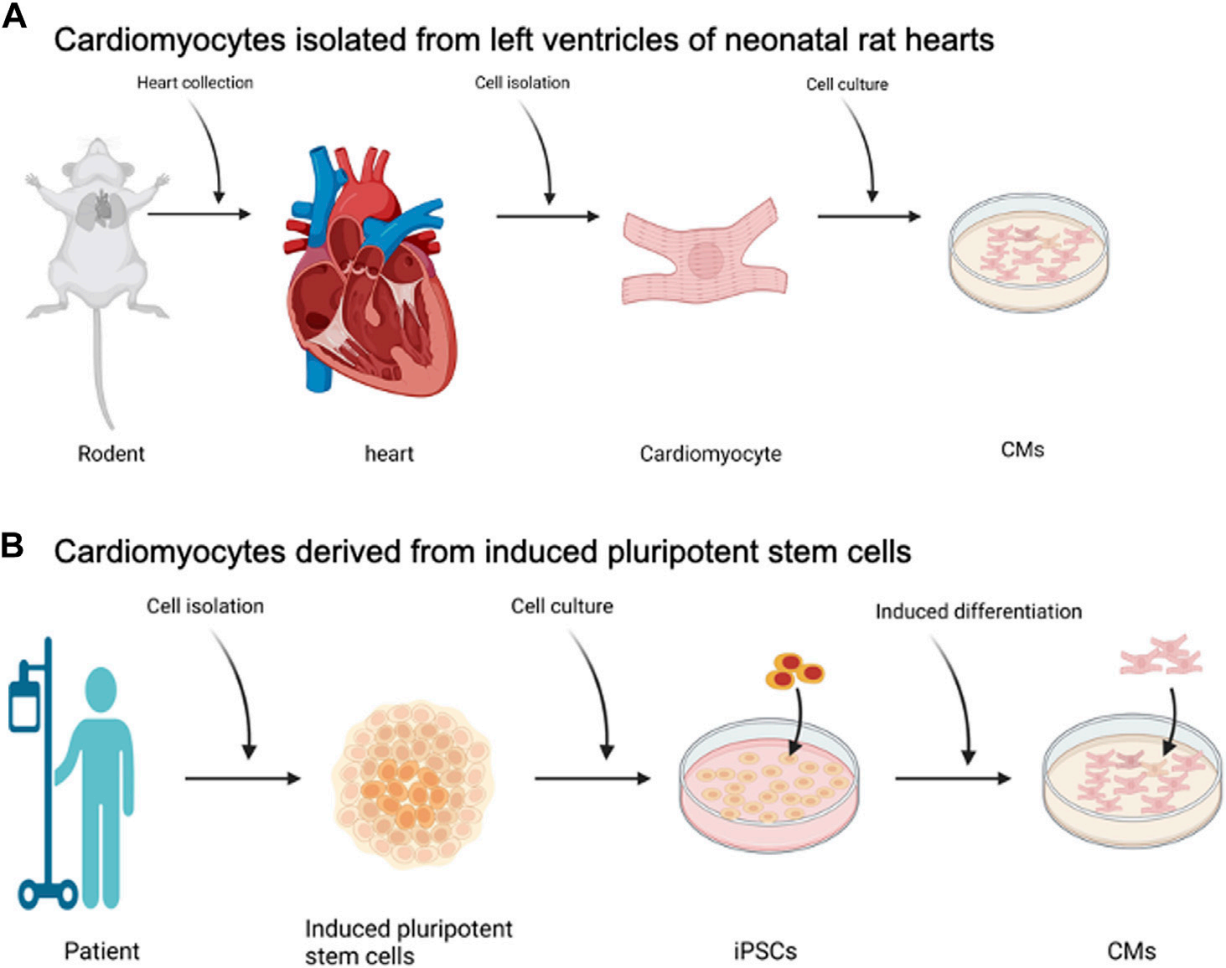
Cell Sourcing for Biological Pumps—**(A)** Cardiomyocytes isolated from left ventricles of neonatal rat hearts, known as NVRMs. **(B)** Cardiomyocytes derived from induced pluripotent stem cells. Created with BioRender.com.

The two cell sources that have been used are primary cardiomyocytes isolated from NVRMs (Khait et al., 2008b) and iPSC-CMs (Zhang et al., 2009; Hattori and Fukuda, 2012; Lian et al., 2013; Tohyama and Fukuda, 2017), Figure 2. The field of biological pumps, and cardiac tissue engineering, started with the use of NVRMs and gradually transitioned to the use of iPSC-CMS. At the time of the inception of the field, iPSC-CMs were not discovered and therefore, not an option for studies; NVRMs were the most practical option during the early stages of the field to support the development of biological pumps. Advantages of NVRMs included ease of availability, low cost, and mature phenotype (Khait et al., 2008b), all of which resulted in outstanding results during the early stages in the development of biological pumps, Figure 2A. NVRMS were particularly advantageous to support the optimization of biological pumps; these studies often required an abundance of cells at high frequency interval, to test the effect of different variables on the formation and function of biological pumps. In addition, the isolation of NVRMS was optimized with standard protocols for isolation and culture (Khait et al., 2008b). These optimization studies were essential to the development of the field and NVRMs provided an excellent tool to support these studies.

The advent of iPSC-CMs created a shift away from NVRMs, Figure 2B. The rationale to do so was the patient derived specificity of iPSC-CMs and the translational potential of these cells (Zhang et al., 2009; Hattori and Fukuda, 2012; Lian et al., 2013; Tohyama and Fukuda, 2017). The primary advantage of iPSC-CMs for the fabrication of biological pumps was to develop personalized solutions that are patient centric. For example, a simple blood draw can be used to isolate peripheral blood mononuclear cells (PBMCs) which are then used to generate iPSCs and iPSC-CMs. This way, the iPSC-CMs are patient centric and specific to the patient. These patient centric iPSC-CMs can then be used to fabricate biological pumps. The key advantage of the biological pumps is the patient centric nature, which means that the functional performance of the biological pump will replicate the pathophysiology of the patient. As a simple example, iPSC-CMs and subsequently biological pumps, can be generated from pediatric patients with hypoplastic left heart syndrome (HLHS), a condition in which a patient is born with a missing or underdeveloped left ventricle. When compared against wild type controls, biological pumps fabricated from HLHS patients can provide insight into changes in the functional performance of these hearts.

The use of iPSC-CMs to fabricate biological pumps is the most practical approach; however, it is not without drawbacks. The use of iPSC-CMs comes with very high cost, highly specialized skills to generate and maintain in culture and the infrequent availability of these cells. These challenges are further confounded by the lack of maturity of iPSC-CMS, which require specialized bioreactors for electromechanical stimulation to support a mature phenotype (Ronaldson-Bouchard et al., 2018).

In addition to electromechanical stimulation, chemical conditioning and changes in the microenvironment have shown to play a key role in driving the maturation phenotype of iPSC-CMs (Huang et al., 2020; DePalma et al., 2021). In most cases, the phenotype of iPSC-CMs still remains immature and much needs to be done prior to generating mature iPSC-CMs that can be used to bioengineer highly functional biology pumps. While NVRMs have been used frequently in the development of biological pumps, these cells are also derived from a neonatal source, and the CMs are less mature then CMs derived from adult sources. Nonetheless, while the immature phenotype of both iPSC-CMs and NVRMs remains a challenge in the field, the former is the clear choice for potential clinical applications while the latter are preferred for initial model development and validation studies.

Working with iPSC-CMs presents itself with another challenge, related to the ability to generate large number of cells at a low cost. With NVRMs, production of a large number of cells is generally not a challenge and this produces a significant advantage to justify the use of these cells. This is particularly true in the case of tissue engineering studies, like the fabrication of biological pumps, which requires hundreds of millions of cells per study. Production of hundreds of millions of iPSC-CMs is yet not feasible in most labs, although recent advances in bioreactors have been developed to push the field in this direction (Hamad et al., 2019). In addition, there have been several recent developments that aim to make iPSC technology more affordable and assess by reducing the cost and frequency of media (Kuo et al., 2020) and simplifying the process to generate iPSC-CMs (Lian et al., 2012). In addition, the utilization of iPSC-CMs for production of biological pumps that can be used clinically will require regulatory challenges and a complex FDA approval process. While these challenges are not specific to biological pumps and are universal to the field of iPSCs as a whole, many scientific, technological and regulatory roadblocks need to be overcome to move the field forward.

The field has shifted towards iPSC-CMS and for the most part, abandoned the use of NVRMs. However, given the current state of the field of biological pumps, where pressure generation in these devices has been reported to be under 5 mmHg, there is still an abundance of rigorous optimization studies required. These studies may be better supported using NVRMs, due to the many advantages described earlier. Progress in the field based on NVRMs can then be translated to iPSC-CMs, providing all the advantages associated with these cells, including personalized medicine.

## Materials for biological pumps

**FIGURE 3:**
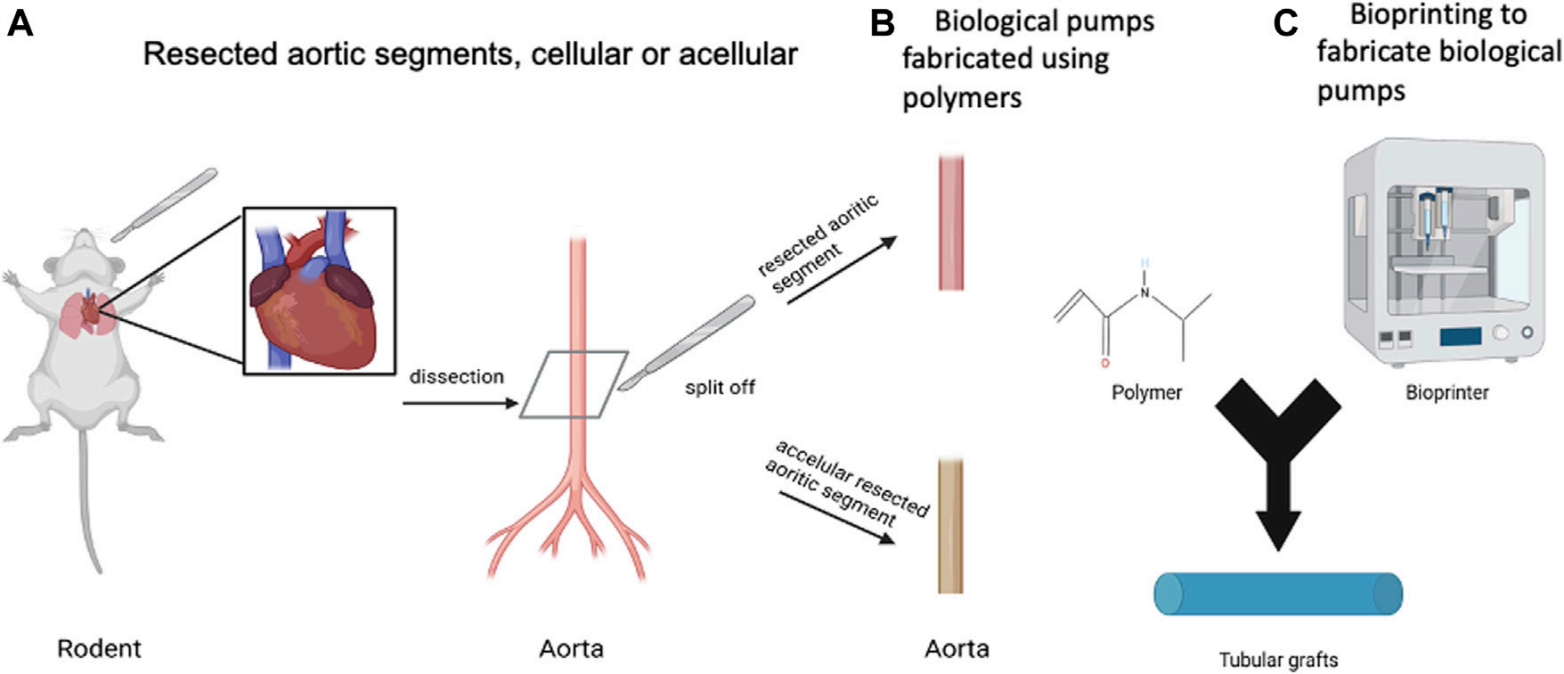
Biomaterials for Biological Pumps—**(A)** Resected aortic segments, cellular or acellular. **(B)** Biological pumps fabricated using polymers. **(C)** Bioprinting to fabricate biological pumps. Created with BioRender.com.

The goal is to fabricate a tubular graft that can be populated with contractile CMs on the outer surface of the graft, Figure 3. There are two main categories of tubular grafts that have been used to fabricate biological pumps, the first based resected samples from rodents, Figure 3A (Sekine et al., 2006) and second, tubular grafts that have been fabricated using molds (Figure 3B) or bioprinting (Figure 3C) (Birla et al., 2008b; Bliley et al., 2022). The result in all cases has been the same, the production of tubular grafts that can support the formation of functional biological pumps.

The primary function of the tubular graft has been to support the repetitive contractions of the CM layer on the outer surface of the graft. In addition to this mechanical role, the tubular graft also has a biological role, to interface with the CMs to support integrin mediated connectivity between the cells and the matrix. This biological role, though critical, has not been demonstrated in any published studies, thereby reducing the role of the tubular graft to support repetitive contractions resulting from the CMs.

There have been examples on the use of resected rat thoracic aorta from rats (Sekine et al., 2006), acellular rat aorta (Birla et al., 2008b), acellular goat carotid arteries (Mohamed et al., 2015), type I collagen (Bliley et al., 2022) and chitosan (Birla et al., 2008b), all of which were effective in generating biological pumps. In most cases, if not all, the studies have been focused on proof of concept and designed to demonstrate functional biological pumps, thought the intra-luminal pressures were always reported to be low. At such low pressures, it is difficult ascertain differences in the performance of the tubular graft, along with relative differences, which will become more apparent as high-performance pumps are generated. Suffice to say, that many different materials have been used to generate biological pumps, all of which have proven to be effective.

## Fabrication methods for biological pumps

While different methods have been used to bioengineer biological pumps, the most common method has been to first fabricate a cohesive monolayer of contracting CMs, and second, physically anchor this cell monolayer to the outer surface of a tubular graft (Birla et al., 2008b; Seta et al., 2017). This approach has been central to several studies and has proven effective in generating functional pumps. While very simple, this approach has worked and continues to be the mainstay of the field. The challenge has been in the development of a cohesive cell monolayer, as well as anchoring to the surface of the tubular graft. Another advantage of this approach is the ability to anchor multiple layers of cell monolayers on the surface of the tubular grafts, thereby providing a mechanism to increase the function of biological pumps.

More recent studies have focused on bioprinting to fabricate biological pumps, with contracting cardiomyocytes suspended in a type I collagen matrix (Bliley et al., 2022). The primary advantage of bioprinting is the ability to program the STL file, the detailed instructions on how to print the biological pump, and load this onto the bioprinter; the machine does the rest. While an interesting approach, bioprinting is still a niche area limited to a few specialized labs.

As the field of biological pumps has progressed, more recent efforts have focused on the use of micro-fluidics to develop “on-the-chip” models of these pumps (Michas et al., 2022). This innovative approach relies on recapitulating the functional characteristics of biological pumps, which include the pressure-volume relationship. However, the goal is to recreate the functional characteristics in a compact chip configuration, rather than recapitulating the anatomy of the biological pump. The smaller footprint allows for the development of high-throughput screening systems, while maintaining the functionality of biological pumps.

## Published methods to fabricate biological pumps

**FIGURE 4:**
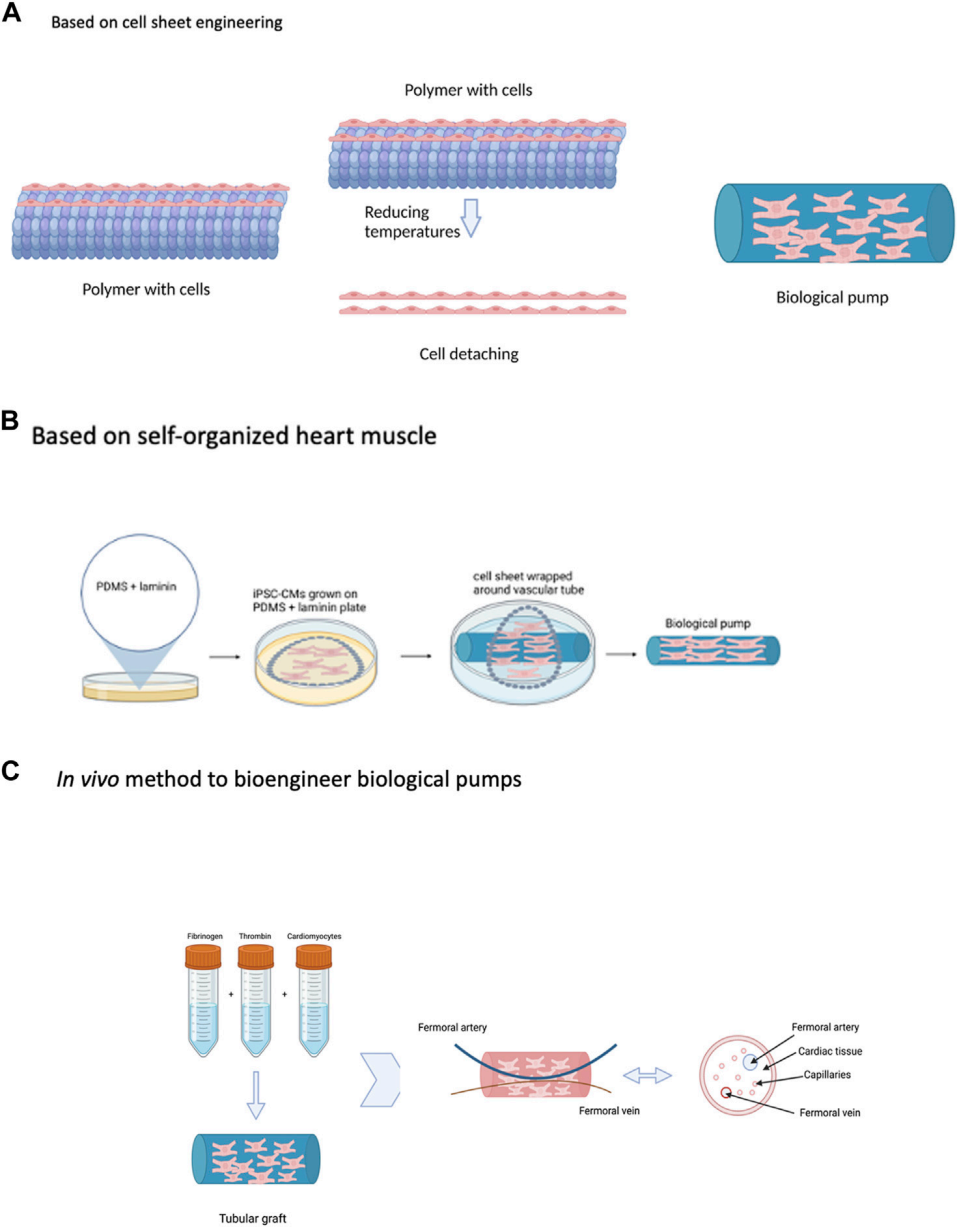
Strategies to Bioengineer Biological Pumps—**(A)** Based on cell sheet engineering. **(B)** Based on self-organized heart muscle. **(C)** In vivo method to bioengineer biological pumps. Created with BioRender.com.

In the previous section, we discussed a general process to bioengineer biological pumps. While this flowchart provides a broad overview of steps that are required to bioengineer biological pumps, not all investigators follow this process, with deviations in the process scheme. Due to the early stage in technology development for biological pumps, many different fabrication methods have been adopted, each with unique advantages and specific challenges. In this section, we provide an overview of many of the methods that have been used to bioengineer biological pumps, Figure 4. This section is designed to provide a survey of current methods to bioengineer biological pumps, and not meant to be an exhaustive list.

### Biological pumps using cell sheeting engineering

This is a novel method to bioengineer pumps and one that has been very influential in the field, Figure 4A (Haraguchi et al., 2014; Sekine et al., 2016; Kobayashi et al., 2019). The central component of this method is the fabrication of sheets of contractile cardiomyocytes, a single layer thick using a novel temperature responsive culture surface (Kobayashi et al., 2019). In this method, tissue culture polystyrene is modified with a thin layer of a temperature response polymer poly(N-isopropylacrylamide) (PIPAAm) (Kobayashi et al., 2019). This modification changes the hydrophobicity of the culture surface as a function of temperature. At 37^o^C, the temperature at which cells are maintained in an incubator, the PIPAAm coated tissue culture surface is hydrophilic and supports the culture and attachment of cardiomyocytes (Kobayashi et al., 2019). Reduction in the culture temperature to 20^o^C changes the surface properties of the PIPAAm from hydrophilic to hydrophobic, resulting in detachment of the cell monolayer (Kobayashi et al., 2019). The unique property of cell sheet engineering, is that the entire cell monolayer detaches as a continuous sheet, maintaining intercellular connectivity. In addition, individual sheets can be stacked to form multi-layer heart muscle tissue, with thickness of two and even three cell layers (Kobayashi et al., 2019). Applied to the fabrication of biological pumps, cell sheets were first generated using primary cardiomyocytes isolated from neonatal rat hearts and cultured on the surface of temperature responsive polymers (Sekine et al., 2006). The resulting cell sheets were physically wrapped around the external surface of thoracic aorta resected from adult rats; a total of 6 individual sheets were layered around the tubular graft (Sekine et al., 2006). Once fabricated, the biological pump was transplanted in place of the abdominal aorta, also in adult rats, for a period of 4 weeks (Sekine et al., 2006). Upon explantation, the biological pumps generated intraluminal pressures more than 5 mmHg (Sekine et al., 2006). The *in vivo* environment produced the physiological environment, in terms of pulsatile conditioning and circulating growth factors and cytokines, to support the maturation of biological pumps. This work was extended to and biological pumps were fabricated using iPSC-CMS, a significant advancement over the use of animal derived cells (Tsuruyama et al., 2019).

The primary advantage of cell sheet engineering to fabricate biological pumps is the remarkable simplicity of the approach, leading to a highly effective and functional pump. The system has been tested and validated with both animal derived cardiomyocytes and iPSC-CMs, providing flexibility in cell sourcing. The ability to generate single layer cell sheets, and cell sheets with two, three and even more layers, provides remarkable customization.

### Biological pumps using self organized heart muscle

This method is based on a novel method to bioengineer 3D heart muscle tissue, one that was developed based on scaffold free technology, Figure 4B (Baar et al., 2005). In the previous method described, cell sheets were fabricated by culturing primary cardiomyocytes on temperature sensitive surfaces; these cell sheets were formed in the absence of any external scaffolding material and served as the scaffold for pump formation. Self-organized heart muscle is based on the same concept, one that makes use of scaffold free methods to bioengineer heart muscle tissue and then make use of this heart muscle tissue as the substrate to bioengineer functional pumps (Birla et al., 2008b). In the case of self-organized heart muscle, the concentration of the adhesion protein, in this case laminin, was used as the guide to tissue formation (Baar et al., 2005). The tissue culture surface is first coated with PDMS, a hydrophobic surface which does not support cell adhesion and growth (Baar et al., 2005). The PDMS surface is then coated with a fixed concentration of laminin, an adhesion protein used to support the culture and growth of primary cardiomyocytes (Baar et al., 2005). An optimized density of primary cardiomyocytes is seeded on the surface of the PDMS/laminin coated plates (Baar et al., 2005). The cardiomyocytes attach to the surface laminin and form a cohesive cell monolayer (Baar et al., 2005). During the next several days in culture, the surface laminin gradually degrades and exposes the cardiomyocytes to the underlying hydrophobic PDMS surface (Baar et al., 2005). This results in detachment of the cell monolayer from the culture surface and subsequent delamination towards the center of the tissue culture plate (Baar et al., 2005). During the delamination process, a pre-fabricated tubular graft is placed in the center and subsequent attached of the delaminating cell monolayer to the tubular graft results in the formation of a biological pump (Birla et al., 2008b). The self-organized heart muscle forms a continuous monolayer of contracting cardiomyocytes surrounding the external surface of the tubular graft (Birla et al., 2008b).

The novelty of this approach is the use of the self-organized heart muscle as a substrate for the fabrication of pumps; the absence of scaffolding material for heart muscle formation resulted in continuous contractions of isolated cardiomyocytes that are transmitted to the underlying tubular graft. This approach has been further extended with biological pumps fabricated using primary smooth muscle cells and skeletal muscle as well (Evers et al., 2011). The use of smooth muscle cells results in high endurance pumps while the use of skeletal muscle cells resulted in high performance pumps.

While cell sheet engineering and self-organization methods are similar, as they both rely on scaffold free technology, there are subtle differences between the two. In the case of cell sheet engineering, changes in temperature are used to guide the formation of cell sheets, while in the case of self-organization, changes in the contraction of the surface adhesion protein are the driving force. The result of both is the same, to create a physiological environment that supports the formation of a cohesive monolayer of contracting cardiomyocytes. From a standpoint of terminology, cell sheet engineering and self-organization has been retained based on the terminology used in the publications that describe these methods.

Cell sheet engineering and self-organization are two very distinct methods to fabricate biological pumps, as discussed before. The functional performance of biological pumps is similar, based on the magnitude of the intraluminal pressure and both methods leading to low pressures. The results are similar for both methods, and this is due to the infancy of both these technologies. The distinction between the two cannot be ascertained at this early stage, though after further optimization, differences may become more apparent based on cellular organization and electrical activity.

### Biological pumps based on extrusion based bioprinting

In this method, an STL file was first created using one of many available software platforms (Bliley et al., 2022); an STL file is a 3D rendering of the object that will eventually be printed, in this case a biological pump. The STL file consists of a set of instructions that guide the mechanical components of the bioprinter and the precise movement of the printhead in the x, y and z-direction. The bioprinter itself, is the central component of the process and there are now many commercially available options, though in this case an in-house custom bioprinter was used (Bliley et al., 2022). Most commercial bioprinters, including the custom bioprinter used in this study are extrusion based, which means the bioink formulation is extruded from the printhead using an external force; in the case of most commercial bioprinters, an external air pressure is used, while in this study, a rotating mechanical screw was utilized (Bliley et al., 2022). The bioink itself is another central component of the process, which consists of a mixture of the biomaterial and the cells and for this study, the bioink consisted of a mixture of type I collagen and iPSC-CMs (Bliley et al., 2022). The final component of this system was the support bath, consisting of microparticles of gelatin (Bliley et al., 2022); the support bath is used as a platform to print into and once the print has been completed, is washed away by an elevation in temperature. The purpose of the support bath was to hold the individual fibers in place during the printing process and increase print resolution. Using iPSC-CMs suspended in type I collagen as the bioprinting, loaded onto a custom fabricated bioprinter, with gelatin microparticles as the support matrix, biological pumps were fabricated (Bliley et al., 2022).

Bioprinting is an interesting strategy to bioengineer biological pumps, as it provides precise control over the geometry of the printed object, offering high resolution. Limitations of this approach are the need for specialized equipment and a high degree of training and technical skill.

### Biological pumps fabricated *in vivo*

**A**ll three methods described earlier are based on *in vitro* technologies, which means that all aspects of the process take place *in vitro*. One of the limitations of all these approaches is the lack of a vasculature, which places an upper limit on the thickness of the cardiomyocyte layer. To overcome this limitation, a very novel method was developed to incorporate vasculature in the cell layer of the biological pump, Figure 4C (Birla et al., 2005); the most important aspect of this method is the use of an *in vivo* biochamber to support the fabrication of biological pumps. In other words, the entire process for the fabrication of biological pumps was conducted *in vivo*; the primary advantage of this was the ability to incorporate a vasculature into the cell layer of the biological pump. In addition, this method was designed in such a way that circulating cytokines and growth factors from the host tissue was utilized to support the development, maturation, and performance of the biological pump (Birla et al., 2005).

NVRMs were suspended in a 3D fibrin gel and placed in the lumen of a silicone tubing, which was then implanted in the groin region of recipient rats (Birla et al., 2005). At the time of implantation, the femoral artery and vein of the host were placed within the silicone tubing, thereby creating a biochamber (Birla et al., 2005). The purpose of the femoral artery and vein was to provide a source for vascularization from the host to the transplanted tissue, provide a source of circulating cytokines and growth factors to promote tissue development and provide a source of pulsatile blood flow to condition the implanted tissue. The implanted tissue was harvested after three weeks, after which time, a biological pump had formed (Birla et al., 2005). The femoral artery and vein were retained as a part of the transplanted tissue, and the transplanted tissue was shown to be vascularized and the intraluminal pressure was reported to be in excess of 2 mmHg (Birla et al., 2005).

The method described in this study is very innovative as it took advantage of an *in vivo* physiological environment to support the formation of a highly functional biological pump. This was also the first time that biological pumps were fabricated using a completely *in vivo* system and was a significant advancement in the field. While this novel *in vivo* system provided a platform for vascularization of biological pumps, it does have limitations. Clearly, an *in vivo* approach will be used primarily for model development and validation studies and not for any work for patient use. In addition, while the in vivo approach described here does take advantage of a complex myriad of signals observed physiologically, these signals are difficult to separate out; as such, the effect of individual compounds on the functional performance of biological pumps cannot be discerned.

## Summary and future perspective

Biological pumps represent a very important field and one where the full potential has yet to be realized. As a standalone device, or as a part of a larger system designed to simulate components of the heart, biological pumps serve an integral component of cardiovascular tissue engineering and represent a cornerstone in the field. While much progress has been made in the field, the functional performance of biological pumps remains low, reported to be less than 5 mmHg; future research needs to target this challenge.

The field has progressed mostly based on iPSC-CMs, while some of the earlier work was conducted using NVRMs. For the field to move forward, a dual strategy needs to be adopted, one which includes the use of NVRMs, as these cells provide a valuable tool for model development and validation. In addition to this, simultaneous work needs to be conducted in generating mature iPSC-CMs, a challenge that is not specific to biological pumps, but the field of stem cell engineering. While progress has been made, using a variety of techniques, including the use of electromechanical stimulation, these methods still do not produce cells with a high degree of maturity and not in the numbers required for the fabrication of biological pumps. In addition, the use of specialized equipment, which is often very expensive, excludes most researchers from using this technology. Furthermore, chamber specific iPSC-CMs exhibit very different functional metrics, for example, with ventricular CMs exhibiting higher forces than atrial CMs (Kane and Terracciano, 2017). Biological pumps fabricated from chamber specific iPSC-CMs are expected to generate very different functional metrics, correlating with the functional metrics of the CMs. This provides a powerful tool to use chamber specific biological pumps to interrogate developmental cues during cardiogenesis and to recapitulate differential functional response of different regions of the heart (ventricle vs. atria).

The scaffolds that have been tested to fabricate biological pumps have been limited; resected aortas, either acellular or with cells, or even the use of chitosan as a scaffold, have not proven to be ideal scaffolds for biological pumps. An ideal scaffold would be one that functionally interacts with the outer cardiomyocytes *via* integrin mediated signaling and that can withstand repetitive contractions over millions of cycles. These criteria have not been met with any of the published scaffolds and is a major challenge that needs to be addressed.

The development of bioreactors is essential for the culture of biological pumps, which are currently maintained in a static environment. Bioreactors are devices designed to simulate the complex *in vivo* milieu during *in vitro* culture and in the case of biological pumps, will consists of coupled electromechanical stimulation. Due to the complexities in bioreactor development and design, only a select few labs can implement this technology in the process development.
